# Assessing alcohol consumption in an era of direct alcohol markers

**DOI:** 10.1016/j.jhepr.2026.101906

**Published:** 2026-06-04

**Authors:** Cecilia Jönsson, Mattias Ekstedt, Patrik Nasr

**Affiliations:** 1 Department of Health, Medicine and Caring Sciences (HMV), Linköping University, Linköping, Sweden; 2 Center for Medical Image Science and Visualization (CMIV), Linköping University, Linköping, Sweden; 3 Department of Gastroenterology and Hepatology, University Hospital, Linköping, Sweden; 4 Wallenberg Center for Molecular Medicine, Linköping University, Linköping, Sweden

**Keywords:** MASLD, MetALD, ALD, PEth

## Abstract

Alcohol-related liver disease (ALD) and metabolic dysfunction–associated steatotic liver disease (MASLD) are the leading causes of advanced liver disease worldwide. The introduction of the steatotic liver disease (SLD) nomenclature, including MASLD, MetALD, and ALD, has increased the need for accurate assessment of alcohol consumption, as disease classification, prognosis, and management depend partly on alcohol exposure. Although structured interviews and validated questionnaires such as AUDIT and AUDIT-C remain standard tools, self-reported alcohol intake is limited by recall bias, underreporting, and variability in drinking patterns. This has led to growing interest in direct alcohol biomarkers that objectively reflect recent alcohol exposure. In this review, we summarise current knowledge on direct alcohol biomarkers, with particular focus on phosphatidylethanol (PEth), the most widely used alcohol marker in hepatology. We conclude that PEth provides a robust and clinically useful measure of recent alcohol consumption, but that its interpretation is influenced by biological variability and context. Importantly, currently used cut-offs are not firmly anchored to clinical outcomes and should not be applied mechanically to classify SLD subtypes. Instead, biomarker-derived measures and self-reported alcohol use should be regarded as complementary tools, best integrated in a longitudinal and clinically contextualised assessment of alcohol exposure.

## Graphical abstract

**Figure:**
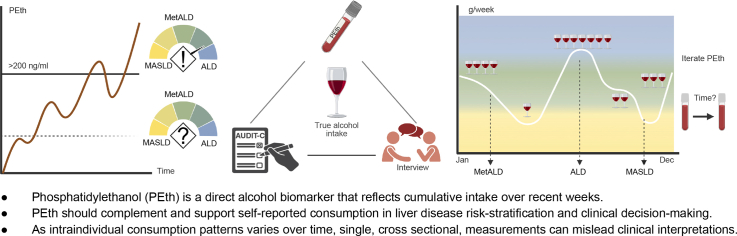

**Keypoints**

• Accurate classification of MASLD, MetALD, and ALD remains limited by systematic bias and underreporting in self-reported alcohol consumption.
• Direct alcohol biomarkers, particularly phosphatidylethanol (PEth), provide an objective and clinically robust assessment of recent alcohol exposure, with clear advantages over indirect markers.
• PEth reflects cumulative alcohol intake over several weeks and is best interpreted longitudinally, using repeated measurements within an appropriate clinical context.
• Reliance on fixed PEth cut-offs to define diagnostic categories is conceptually flawed, as it imposes arbitrary thresholds on a continuous exposure-risk relationship. PEth is better used quantitatively to support risk stratification and clinical decision-making.
• Although PEth concentrations above ∼200 ng/ml are commonly considered indicative of high-risk alcohol consumption, lower thresholds intended to define abstinence or moderate intake should be viewed as pragmatic reference values rather than definitive diagnostic cut-offs.
• Direct biomarkers should complement, not replace, structured clinical assessments, including interviews and validated instruments such as AUDIT-C.
• PEth should be integrated into routine clinical practice and clinical trials to enable more accurate phenotyping, improved patient stratification, and more reliable evaluation of treatment effects.

## Introduction

Alcohol-related liver disease (ALD) and metabolic dysfunction–associated steatotic liver disease (MASLD) have become the dominant causes of advanced liver disease globally.[1], [2], [3], [4] Previously, steatotic liver disease (SLD) was classified as alcohol-related or non-alcohol-related fatty liver disease, with the latter requiring exclusion of significant alcohol intake using strict alcohol consumption cut-offs. This distinction relied largely on self-reported alcohol use, obtained either through clinician-led interviews or questionnaires such as the Alcohol Use Disorders Identification Test (AUDIT).[5], [6], [7] In clinical practice, it is often difficult to apply such a dichotomous distinction between the presence and absence of excessive alcohol consumption. Drinking patterns change throughout the year (*e.g*. some individuals increase consumption during holidays) and across the lifespan (*e.g.* some reduce drinking after having children or increase consumption following major life events), while self-reporting is inherently prone to recall bias and underreporting.^8^

With the introduction of the new nomenclature, this binary classification was replaced by graded subcategories including MASLD, metabolic and alcohol-related liver disease (MetALD), and ALD. With this shift, greater emphasis was placed on the importance of accurately quantifying daily and/or weekly alcohol consumption to help distinguish between these entities, which are defined by thresholds of weekly intake, with cut-offs of 210/140 g for men/women separating MASLD from MetALD, and 420/350 g for men/women distinguishing MetALD from ALD.^9^ This discrimination is required not only for academic or scientific purposes, but more importantly to tailor treatment and follow-up, as rates of disease progression differ across subgroups.^10^^,^^11^ However, the limitations of self-reported alcohol consumption remain.[12], [13], [14]

Given the challenges of self-reported alcohol intake, objective biomarkers have become increasingly important in both research and clinical practice.^15^^,^^16^ Conventional indirect markers of alcohol use, such as mean corpuscular volume, gamma-glutamyl transferase, and carbohydrate-deficient transferrin (CDT), lack sufficient sensitivity and specificity, which has limited their use.^17^ To address these limitations, direct alcohol biomarkers have emerged as promising tools for detecting and quantifying recent alcohol consumption.^18^^,^^19^ These markers, including phosphatidylethanol (PEth), ethyl glucuronide (EtG), and ethyl sulfate (EtS), are formed as specific metabolites of ethanol.^20^ They provide a more objective measure of alcohol exposure, with varying detection windows and biological matrices available for testing, such as blood, urine, and hair.^19^ In recent years, PEth in particular has attracted increasing attention in hepatology, owing to its high specificity and a somewhat linear dose-response relationship.^21^

The application of direct alcohol markers in the setting of SLD may offer diagnostic and clinical advantages.^10^^,^^11^^,^^21^^,^^22^ They can help distinguish ALD from MASLD, provide insight into patterns of drinking behaviour, and support monitoring of abstinence or reduction of alcohol consumption in patients with established liver injury.^23^^,^^24^ Moreover, their integration into clinical practice and research could improve patient stratification in clinical trials and enable more personalised management strategies.^21^^,^^25^

In this review, we summarise the current evidence on direct alcohol markers, focusing on their analytical performance, clinical utility, and potential role in the management of SLD. We also highlight limitations, remaining knowledge gaps, and future directions for their implementation in hepatology.

## Burden of alcohol consumption

**Fig. 1:**
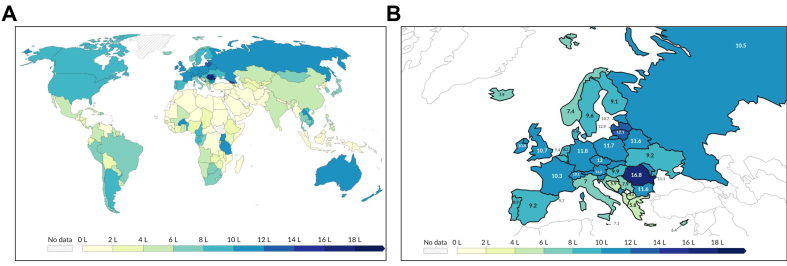
Global trends in alcohol consumption. Annual average alcohol consumption per person (aged ≥15 years, 2020), both globally (A) and in Europe (B). To account for different alcohol contents among alcoholic drinks (*e.g*. beer, wine, spirits), quantities are expressed in litres of pure alcohol per year (ourworldindata.org/grapher/total-alcohol-consumption-per-capita-litres-of-pure-alcohol).

In 2020, the global level of annual alcohol consumption in individuals aged 15 years and over was 4.9 L of pure ethanol per person, representing 1.3 standardised units of alcohol (*i.e*. 10 g/unit) per day (Fig. 1A). Substantially higher numbers are recorded in Europe (10.2 L pure ethanol/year) and North America (9.9 L pure ethanol/year), representing 2.8 and 2.7 standardised units of alcohol per day (Fig. 1B). In Europe, the proportion of individuals who had abstained from alcohol during the previous year ranged from approximately 20% to 60%, with most countries reporting values just above 20%. Consequently, alcohol consumption among drinkers is considerably higher than population-level estimates suggest. For example, given that 22.5% of Swedish adults report being abstainers, the average consumption among drinkers would be approximately 12.5 L of pure ethanol/year, compared with the population average of 9.6 L.

The global proportions of drinkers and abstainers have remained relatively stable over time, but the consumption level of 2020 represents a reduction relative to the level observed in 2019, following nearly a decade of minimal change.^26^ Between 2010 and 2019, global per capita consumption fell by less than 5% (from 5.7 L to 5.5 L). By contrast, from 2019 to 2020 consumption dropped by 10%, likely owing to the COVID-19 pandemic.^27^^,^^28^ Despite reductions at the population level, alcohol consumption among drinkers remains high and the alcohol-related health burden is increasing.^29^^,^^30^ In 2021, there were over 111 million cases of alcohol use disorder, 3 million cases of ALD, and 130,000 cases of alcohol-attributable liver cancer, corresponding to increases of 14.7%, 38.7%, and 94.1%, respectively, since 2000.^30^

## Alcohol metabolism

**Fig. 2:**
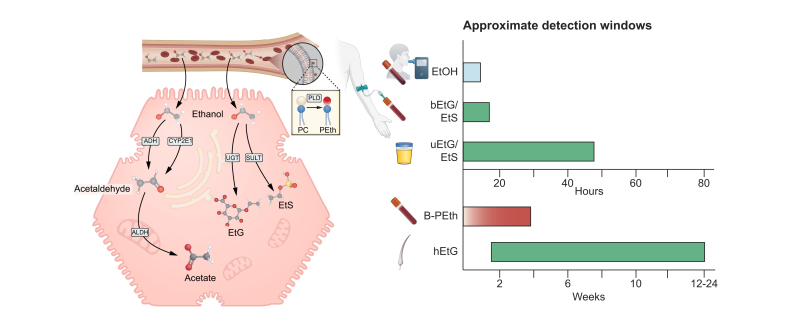
Ethanol metabolic pathways and detection windows of direct biomarkers across matrices. Ethanol is primarily metabolised in hepatocytes through oxidative pathways. The major route involves its conversion to acetaldehyde by ADH and CYP2E1. Acetaldehyde is subsequently oxidised to acetate by ALDH, after which acetate is released from the liver. A smaller fraction of ethanol undergoes non-oxidative metabolism. In these pathways, ethanol is conjugated with glucuronic acid or sulfate by UGT and SULT, forming EtG and EtS, respectively. PLD, broadly expressed in and outside hepatocytes, forms PEth by transferring a phosphatidyl group to ethanol. A characteristic feature of PEth metabolism is that erythrocytes lack enzymes to degrade it, enabling its stable accumulation in their membrane. Ethanol can be detected directly in blood or breath for up to ∼12 h, reflecting recent alcohol intake. EtG/EtS extends this detection window: and can be measured in blood for ∼15 h and in urine for ∼48 h after consumption. PEth in blood and EtG in hair provide even longer detection windows. PEth can remain detectable for up to 4 weeks after the last alcohol consumption, while EtG in hair can reflect alcohol intake in the preceding 3–6 months, depending on hair length and with a lag time of 1–2 weeks before the marker appears in the hair shaft. ADH, alcohol dehydrogenase; ALDH, aldehyde dehydrogenase; CYP2E1, cytochrome P450 2E1; EtG, ethyl glucuronide; EtS, ethyl sulfate; PEth, phosphatidylethanol; PLD, phospholipase D; SULT, sulfotransferase; UGT, UDP-glucuronosyltransferase.

Following alcohol intake, approximately 90% of ingested ethanol undergoes oxidative metabolism to acetaldehyde, primarily within the liver. The primary enzyme in oxidative metabolism is alcohol dehydrogenase (ADH), while cytochrome P450 2E1 (CYP2E1) plays a secondary role (Fig. 2).^31^ Because CYP2E1 has a much lower affinity for ethanol than ADH, its contribution becomes significant mainly during heavy or binge drinking.^32^^,^^33^ Because ethanol is cleared quickly, usually within 15 h, measurement of blood or breath alcohol levels only reveals very recent intoxication.^34^^,^^35^ To detect alcohol consumption over longer periods, direct ethanol-derived metabolites formed through minor, non-oxidative pathways (about 5% of total ethanol metabolism) are measured. These metabolites include PEth, EtG, EtS, and fatty acid ethyl esters (FAEEs) (Fig. 2).^19^ PEth is formed in the presence of ethanol when a phosphatidyl group from phosphatidylcholine is transferred to ethanol.^36^^,^^37^ EtG and EtS are produced when ethanol is conjugated with endogenous glucuronic acid and sulfate, while FAEEs arise through enzymatic esterification of ethanol with free fatty acids, triglycerides, lipoproteins, or phospholipids.^19^ Each of these metabolites can serve as markers of alcohol consumption, offering different detection windows, ranging from hours to weeks, depending on both the biomarker and the sample type (Fig. 2).

## Assessing alcohol consumption

### Interview

Assessing alcohol consumption is challenging since no single assessment sufficiently captures the complexity of alcohol use.^12^^,^^13^ To capture long-term patterns of alcohol use that extend beyond recent consumption, the Lifetime Drinking History has been developed as a retrospective, interview-based assessment designed to identify patterns of alcohol use, abuse, and dependence across the lifespan, beginning with the onset of regular drinking and continuing through to the individual’s current drinking pattern.^38^

However, the most common approaches in clinical and research settings rely on self-reported measures, such as the TLFB (timeline followback) method for quantitative drinking estimates, using calendar-based cues to reconstruct recent alcohol use, and the AUDIT to screen for hazardous drinking.[39], [40], [41] The AUDIT has 10 questions that explore consumption (Q1-3), dependence (Q4-6), and alcohol-related problems (Q7-10).^40^ The most commonly used tool for excluding excessive alcohol consumption is the AUDIT-Consumption (AUDIT-C) questionnaire, which includes only the first three questions of the AUDIT.^42^

Since average alcohol consumption levels in Europe substantially exceed the levels typically self-reported in clinical care, concerns arise regarding both the clinician’s ability to elicit an accurate alcohol history and the patient’s ability to recall and report alcohol use reliably.[12], [13], [14]^,^^43^ Consequently, relying solely on AUDIT or AUDIT-C may lead to underestimation of alcohol consumption. In a recent study of 2225 middle-aged adults in Sweden, AUDIT-C identified the majority of individuals with PEth-defined excessive alcohol consumption; however, 26% were still classified as low or moderate consumers.^44^

Although validated questionnaires such as AUDIT(-C) are useful, recall bias and underreporting remain important limitations.

### Indirect markers

In addition to AUDIT(-C), clinicians sometimes use indirect alcohol markers, such as gamma glutamyltransferase, mean corpuscular volume, aspartate aminotransferase, and alanine aminotransferase to guide the interview. However, these biomarkers primarily reflect sustained heavy drinking and are influenced by multiple confounding factors, limiting their sensitivity and specificity; they should therefore not be used as standalone markers of alcohol consumption.[45], [46], [47], [48], [49] A more specific, though still indirect, alcohol marker is CDT.^50^ CDT mainly indicates heavy alcohol intake (>50–80 g/day or 350–560 g/week for >1–2 weeks) – a threshold far above the MASLD limit, and closer to the definition of ALD. Like other indirect alcohol markers, CDT remains susceptible to confounding comorbidities, resulting in clinically relevant misclassification and limiting its reliability as a marker of alcohol consumption.[51], [52], [53]

### Direct alcohol markers

The metabolites formed through non-oxidative ethanol metabolism function as direct markers of alcohol intake.^19^ These compounds can be quantified in blood, with detection windows of up to approximately 4 weeks for PEth, ∼15 h for EtG and EtS, and 24–44 h for FAEEs.[54], [55], [56], [57], [58], [59] EtG and EtS may also be measured in urine, where their detection windows extend to approximately 24–48 h after moderate drinking and up to several days following heavy consumption.^60^^,^^61^ Together, these matrices enable detection of recent alcohol intake, capturing consumption from the preceding hours to several days, while PEth additionally reflects sustained intake over several weeks. For even longer retrospective assessment, EtG and FAEEs incorporated into hair provide detection windows spanning several months.[62], [63], [64] Among these direct alcohol markers, PEth has emerged as a leading biomarker in liver disease.^65^ Its concentration correlates strongly with alcohol consumption and, as a blood-based marker, it can be readily integrated into clinical workflows while offering the longest detection window available in blood. Though studies have shown high accuracy and few confounding factors, the exact association between alcohol consumption patterns and PEth dynamics is still unclear.

In the United States, a cut-off of 20 ng/ml (0.028 μmol/L) for PEth 16:0/18:1 is widely used to differentiate deliberate alcohol consumption from abstinence or incidental exposure, as most commercial laboratories report a test as positive only at ≥20 ng/ml, which functions as the minimum reporting level.[66], [67], [68] In Sweden, a higher cut-off of 35 ng/ml (0.05 μmol/L) is applied, reflecting national harmonisation guidelines.^69^

### Biogenesis, distribution, and clearance of PEth

PEth is formed exclusively in the presence of ethanol, when phospholipase D (PLD) catalyses a reaction that transfers a phosphatidyl group from phosphatidylcholine to ethanol.^36^^,^^37^ Because ethanol is favoured over water as a substrate, PLD produces PEth instead of its normal product, phosphatidic acid, and the amount generated increases with ethanol concentration. Following alcohol intake, PEth can be detected in several organs and in blood, where it is found almost exclusively within the erythrocyte fraction.^70^^,^^71^ Since erythrocytes lack the enzymes required to degrade PEth, the compound remains in these cells with a relatively long half-life compared to other cell types.^72^

PEth is not a single molecule but a class of phospholipids with a phosphoethanol head group and two fatty acid chains attached, with variable length and saturation. Among the 48 identified variants, PEth 16:0/18:1 (containing one palmitic acid [16:0] and one oleic acid [18:1]) and PEth 16:0/18:2 (containing one palmitic acid and one linoleic acid [18:2]) are the predominant species, accounting for approximately 37–46% and 26–28% of total PEth, respectively, both showing strong correlations with total PEth.^19^^,^^67^^,^^73^ Among these, PEth 16:0/18:1 typically reaches the highest concentrations in blood and is the most sensitive biomarker of alcohol consumption.^74^ It is therefore the preferred analyte in most routine clinical methods.^66^^,^^75^^,^^76^

PEth is not subject to enzymatic degradation, and its elimination depends primarily on erythrocyte turnover and the gradual renewal of membrane phospholipids.^77^ Most studies report a PEth half-life of 4 to 10 days; however, it has been detected in patients undergoing withdrawal treatment for alcohol use disorder even after more than 6 weeks of abstinence.[54], [55], [56]^,^^78^ Importantly, because PEth is eliminated slowly, it accumulates with repeated drinking episodes, making drinking patterns a major determinant of PEth levels. Although five half-lives are often used as a rule of thumb for drug elimination, this provides only an approximate estimate for PEth clearance, and the commonly stated detection window is about 1 month.^79^

### Modulators of PEth concentration

PEth formation is directly linked to blood alcohol concentration (BAC), and any factor that alters alcohol absorption or distribution affects PEth production. Eating before or during alcohol consumption slows gastric emptying and reduces peak BAC.^80^ Peak BAC depends on the amount of total body water available for alcohol dilution. In theory, individuals with higher body weight reach lower BAC levels after an equivalent alcohol dose. However, two people with identical body weight but different BMI may reach different BAC values because differences in total body water^81^ influence PEth formation, leading to under- or over-estimation of alcohol intake. Women typically have less total body water than men, reach a higher BAC and could theoretically produce more PEth. Although no sex-related differences in PEth formation or elimination kinetics have been demonstrated, sex has been found to independently predict alcohol consumption at a given PEth concentration.^82^^,^^83^ BAC clearance depends on oxidative ethanol metabolism, and heavy drinking can upregulate CYP2E1, accelerating BAC clearance and potentially reducing PEth formation.^32^^,^^84^^,^^85^ Whether ADH adapts to chronic alcohol exposure is less clear, as studies report conflicting results.^31^^,^[86], [87], [88]

As PEth is incorporated into erythrocyte membranes, conditions that shorten red cell lifespan, such as haemolysis, could cause disproportionately low PEth despite alcohol consumption. Transfusion of packed red blood cells can transiently increase concentrations through the introduction of donor-derived PEth.^89^^,^^90^ Other rare or incidental sources of ethanol may also influence PEth formation. Certain gut microbes can produce significant amounts of endogenous ethanol, as seen in auto-brewery syndrome.^91^ While unstudied, substantial endogenous ethanol generated in these rare cases could theoretically increase PEth concentrations. However, the modest endogenous ethanol shown to be produced in SLD is insufficient to elevate PEth concentrations above clinical decision thresholds.^92^ Likewise, incidental ethanol exposure from products such as mouthwash or hand sanitizers does not raise PEth values to levels that could be misinterpreted as intentional alcohol consumption.[93], [94], [95], [96]

### Analytical methods for PEth detection

The breakthrough of PEth as a clinical biomarker came with the development of advanced analytical methods like liquid chromatography–tandem mass spectrometry (LC–MS/MS). Initially discovered using thin-layer chromatography, PEth’s application was limited by poor sensitivity and throughput.^36^ High-performance liquid chromatography with evaporative light scattering improved quantification but lacked resolution for structural heterogeneity.^97^ LC–MS/MS increased sensitivity more than 100-fold and enabled rapid, homologue-specific analysis.^73^^,^^98^

Despite its specificity, PEth measurement still faces challenges, particularly inter-laboratory variability arising from differences in extraction procedures and solvents, which may lead to different classifications of alcohol consumption.^99^ To address this, methods are being refined through optimised extraction protocols, high-throughput LC–MS/MS workflows and emerging techniques such as supercritical fluid chromatography–MS/MS.[99], [100], [101], [102] Ensuring analytical validity and transparent reporting is critical. The 2022 Basel Consensus provides standardised recommendations for clinical and forensic use,^67^ and routine implementation in Sweden, supported by an external quality assessment programme, illustrates how harmonised decision limits and inter-laboratory comparisons can reduce variability (from >20% coefficient of variation in 2018 to ∼12% in 2021–2022) and strengthen clinical confidence.^75^

### Drinking patterns and PEth

The cut-offs used for PEth 16:0/18:1 (hereafter ‘PEth’) are derived from clinical experience in combination with the analytical detection capabilities of the assays, rather than from the relationships between PEth concentrations and quantified alcohol consumption.^103^ Efforts have been made to investigate the lowest amount of alcohol intake required to exceed a PEth level of 20 ng/ml in controlled study environments. In one study by Stöth *et al.* of 12 individuals (6 men and 6 women), consumption of a single alcoholic drink (19–45 g alcohol) designed to reach a BAC of 0.5 g/kg did not produce detectable levels of PEth.^104^ In contrast, another study of nine volunteers (4 men and 5 women) found that one drink containing 34–72 g of alcohol (targeting a BAC of 1 g/kg) resulted in PEth levels ranging from 35–120 ng/ml.^105^ However, unlike the study by Stöth *et al.*, where adherence to the preceding 4-week abstinence period was confirmed by PEth results <20 ng/ml in all participants, five of the nine participants in the latter study had baseline PEth levels between 20 and 30 ng/ml after the required 2-week abstinence period. Additional evidence comes from a study of 75 individuals who, after a confirmed baseline PEth value <7 ng/ml, consumed 20 g of alcohol per day for three consecutive evenings. After the first day, 1% of participants reached a PEth concentration >20 ng/ml, and after 3 days, 7% exceeded 20 ng/ml, of whom 1% exceeded 35 ng/ml.^106^ A notable difference compared with the study by Stöth *et al.* is the sex distribution: 72% of participants were female, and all individuals with a PEth >20 ng/ml were women. Although there are interindividual differences in PEth formation, consuming 20–40 g of alcohol on one or a few occasions following a period of abstinence is unlikely to produce PEth values >20 ng/ml in most individuals. A PEth value below this cut-off is generally considered compatible with abstinence or low alcohol consumption.^67^^,^^107^

A longer consumption period was evaluated by Walther *et al.* and Kechagias *et al.*^103^^,^^108^ Walther *et al.* assigned 21 individuals to either one glass of wine daily (*i.e*. 16 g of alcohol) or two glasses of wine daily (*i.e.* 32 g). After 2 weeks, mean PEth levels were 17 ng/ml (range 7–30 ng/ml) and 37 ng/ml (range 6–65 ng/ml) in the respective groups.^103^ In the study by Kechagias *et al.*, 44 individuals were randomised to abstinence or daily consumption of 16 g (female) or 32 g (male) of alcohol (wine) for 12 weeks.^108^ Most participants in the alcohol group had PEth values <35 ng/ml, though three reached levels of 49, 84 and 119 ng/ml.^108^ The studies by Kechagias and Walther differ in several important aspects. The study by Kechagias *et al.* included a higher proportion of female participants compared with the study by Walther *et al.* (approximately 70% *vs.* 50%). Although sex distribution alone would not be expected to substantially influence PEth levels, women in the Kechagias study consumed approximately half as much alcohol (16 g) as men, which may have contributed to lower PEth concentrations. In addition, Kechagias *et al.* did not perform an interim PEth analysis to verify adherence to the alcohol consumption protocol. Taking these differences in study protocols into consideration, both trials nonetheless yielded comparable results, with mean PEth concentrations at or below approximately 35 ng/ml in individuals consuming 16–32 g of alcohol per day.

In a non-controlled study environment, Finanger *et al.* asked 62 study participants to register their alcohol consumption.^109^ On average, those with self-reported alcohol consumption equivalent to two units per day did not exceed a PEth value of 70 ng/ml. Similarly, a study by Helander *et al.* showed that an increase in consumption equivalent to 1.5 drinks per day was associated with an increase in PEth of approximately 70 ng/ml, with a comparable decrease observed for reductions in intake.^110^ Concordantly, in another study, 222 patients with chronic liver disease had PEth analysed and compared to patient-reported alcohol use through the TLFB method.^111^ The study proposed a threshold of ≥80 ng/ml for identifying daily consumption of ≥4 drinks/day (≥56 g/day), with sensitivity and specificity of 91% and 77%, respectively.

These studies suggest that, in controlled study settings, consumption of up to 30 g of alcohol per day rarely leads to PEth levels >35 ng/ml, whereas in non-controlled settings relying on self-reported intake, reported consumption of “two glasses per day” rarely results in PEth levels >70 ng/ml. The PEth threshold of 70–80 ng/ml is supported by data from Torp *et al.*, showing that in a large cohort of 2,924 individuals, PEth levels >80 ng/ml were uncommon among those with low self-reported alcohol consumption or low AUDIT-C scores.^22^

Thus, despite some discrepancies between controlled and non-controlled studies, available data suggest that individuals with PEth concentrations below approximately 70–80 ng/ml generally report alcohol consumption of two standard drinks per day (in self-reported settings), or around 30 g of alcohol per day (in controlled studies), sustained over longer periods. These levels are consistent with the proposed upper threshold for MASLD. Accordingly, PEth values exceeding ∼80 ng/ml may be more compatible with MetALD or ALD, although such categorisation should be regarded as indicative. Based on findings by Torp *et al.*, a PEth concentration of approximately 200 ng/ml may represent a reasonable level of ALD.^22^ This threshold is also in agreement with the Consensus of Basel^67^ and broadly aligned with definitions of excessive alcohol consumption (∼210 ng/ml) according to Swedish national guidelines and German driving license re-granting regulations.^112^

### Liver disease and PEth

In patients with liver disease, impaired liver function could prolong elevated blood alcohol levels, alter ethanol metabolism, and complicate biomarker interpretation. Nevertheless, available evidence suggests that PEth remains a reliable biomarker in early liver disease.^113^^,^^114^ While simple fibrosis appears to have little impact, cirrhosis creates a dynamic interplay of mechanisms that challenge interpretation. Enzyme activity studies have shown that ADH activity is largely preserved in chronic drinkers and non-cirrhotic liver disease but decreases in cirrhosis regardless of aetiology.^88^ CYP2E1-mediated drug metabolism was also found to be impaired in patients with ALD only when cirrhosis was present.^115^ These mechanisms of impaired alcohol metabolism would increase PEth levels. However, a study of 100 heavy drinkers during inpatient detoxification highlighted a nuanced impact of cirrhosis on alcohol biomarkers.^113^ Participants with cirrhosis demonstrated higher serum levels of alcohol, EtG, and EtS compared to participants without cirrhosis despite comparable alcohol intake. PEth concentrations were also elevated, although not significantly so, likely reflecting increased erythrocyte turnover.^113^^,^^116^ A recent study also reported that PEth values correlated with Child-Pugh scores but not with anaemia, BMI, or sex in a cohort with alcohol-related cirrhosis; however, most participants were Child-Pugh class A or B. These findings suggest that PEth levels in cirrhosis are affected by opposing physiological processes. Prolonged systemic alcohol exposure from impaired hepatic clearance, altered portal–systemic blood flow, and reduced renal excretion can lead to unexpectedly high PEth values.^117^ In contrast, haemolytic anaemia and reduced erythrocyte production from impaired hematopoietic stem cell activity may lower PEth by shortening red cell lifespan.^118^ These opposing influences underscore the importance of interpreting PEth results within the broader clinical context and highlight that PEth should not be used in isolation to assess alcohol use in patients with liver disease.

### Other direct markers

Beyond PEth, the direct ethanol metabolites EtG, EtS and FAEEs are used for detecting alcohol consumption across multiple biological matrices. Given their short detection windows in blood, EtG and EtS are predominantly determined in urine. In clinical practice, urine EtG testing is used mainly for monitoring abstinence during alcohol detoxification and in transplant settings, where it complements self-reports in cases of suspected underreporting and is sometimes combined with PEth testing.^119^^,^^120^ Urine EtS is more stable and less vulnerable to degradation or microbial interference, whereas EtG is typically present at higher concentrations and detectable for a slightly longer period; therefore, EtS is most often measured to confirm EtG results.[121], [122], [123], [124] However, the added value of EtG/EtS testing compared to PEth alone has recently been questioned.^125^ EtG analysis in 3-6 cm proximal head hair segments reflects alcohol consumption during the previous 3 to 6 months, and the Society of Hair Testing has established hair EtG cut-off values to support the assessment of abstinence and chronic excessive drinking.^62^^,^^64^ However, since hair takes about 1–2 weeks to grow out of the scalp, alcohol use during the two weeks prior to sampling may not be captured.^126^ Hair EtG is mainly used in forensic contexts, but is also applicable clinically, including liver transplant eligibility, due to its long detection window and stability across age, sex, and liver disease.^62^ Nonetheless, its sensitivity to cosmetic treatments, external contamination, and methodological variability necessitates standardised protocols and careful control of confounders.^115^ Hair collection may also be limited by alopecia, male pattern baldness and short hair, or in individuals who routinely shave, such as athletes. In recent years, nails have emerged as a promising alternative matrix for EtG analysis, often producing higher EtG concentrations than hair and providing detection windows of approximately 2–3 months post consumption.^63^^,^^127^ However, the lack of standardised cut-offs and interpretation guidelines, along with variability in nail growth and the potential for external contamination, still limit their widespread application.^128^

FAEEs comprise a group of over 20 non-oxidative ethanol metabolites.^129^ FAEEs are detectable in blood plasma and hair; however, FAEE concentrations do not seem to correlate linearly with the amount of alcohol consumed and are primarily used to determine whether heavy drinking has occurred.^17^ The Society of Hair Testing currently recommends ethyl palmitate as the sole FAEE marker, with two cut-off values for abstinence and chronic excessive drinking.^64^ Nevertheless, ethyl palmitate has limited diagnostic specificity due to its susceptibility to false positives from the use of ethanol-containing hair products. Consequently, it is not recommended as a standalone biomarker for the assessment of abstinence.^64^

### Role of past and present alcohol consumption

In contrast to other lifestyle exposures such as smoking, where cumulative exposure can be quantified using the well-established tobacco “pack-year” metric, no standard measure exists for cumulative alcohol exposure over time.^130^^,^^131^ In both research and clinical practice, alcohol use is commonly assessed through self-reported recall of average daily consumption, which informs treatment decisions, trial inclusion, and risk prediction. Depending on the assessment method, these estimates may reflect intake over the preceding 12 months (*e.g*. AUDIT), the past 1–3 months (clinical interviews), or only the previous couple of weeks when using PEth. This limitation is particularly relevant in SLD, where the thresholds for MASLD, MetALD, and ALD are based on a cross-sectional estimation of consumption rather than lifetime drinking patterns. The use of Lifetime Drinking History is a reliable and validated way of assessing consumption patterns during different eras of an individual’s life in a research setting.^131^ However, in our experience, when using the Lifetime Drinking History questionnaires in a clinical setting, patients often perceive it as tedious and difficult to answer – with recall bias being the main issue.

Given these constraints, it is necessary to use the available tools in the most careful and integrated manner possible to achieve the most reliable estimate of actual alcohol consumption. Using all three approaches (i.e., clinical interview, AUDIT-C and PEth) in combination, effectively “triangulating” consumption to reach a consensus, likely represents the most robust method for estimating recent consumption patterns, particularly over the past month. This approach is further strengthened when PEth measurements are repeated.

**Fig. 3:**
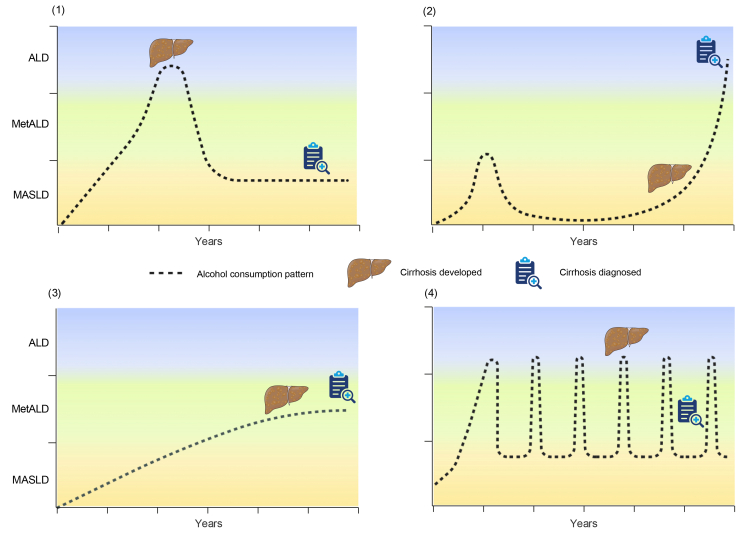
Illustrative scenarios demonstrating how lifelong drinking patterns influence both the timing of cirrhosis development and the aetiology assigned at diagnosis. Four trajectories are shown: (1) gradually increasing to heavy alcohol intake over several years followed by low consumption, (2) early moderate drinking followed by long-term abstinence and a later major increase in consumption, (3) gradually increasing to moderate alcohol intake over several years, and (4) intermittent “holiday drinking” with high-intensity consumption during specific periods. ALD, alcohol-related liver disease; MASLD, metabolic dysfunction-associated steatotic liver disease; MetALD, metabolic dysfunction-associated alcohol-related liver disease.

Real-world data further illustrates the temporal variability of alcohol consumption. In a recent Swedish primary care study by Månsson *et al.*, including 110,447 PEth values from >60,000 patients, the prevalence of hazardous alcohol use (defined as PEth >210 ng/ml) was higher during summer months, peaking in July (16%), compared with November (11%).^132^ Also, as shown in a prospective study by Israelsen *et al.* including 595 individuals with SLD, the classification of SLD changed in approximately 30% of participants during a 2-year follow-up.^133^ Against this background, an individual presenting with decompensated cirrhosis in their sixties may have abstained from alcohol for years or even decades yet still have had substantial alcohol exposure earlier in life, for example during their twenties and thirties. In such individuals, compensated cirrhosis may have developed later, for instance in their forties, followed by prolonged abstinence before eventual decompensation. Despite this remote history of heavy alcohol use, the current disease phenotype, based on recent alcohol exposure, would likely be classified as MASLD (Fig. 3). Conversely, an individual who develops hepatic decompensation after a relatively short period (*e.g.* 6–12 months) of high alcohol consumption, despite a longstanding history of obesity and metabolic risk factors, may be classified as ALD rather than MetALD (Fig. 3). Such differences in classification may have important clinical implications, including on transplant waiting time and the risk of further liver-related complications.

## Conclusion

Since the introduction of the revised nomenclature, research activity in MASLD, MetALD, and ALD has increased substantially. Stratification of SLD according to levels of alcohol consumption has highlighted alcohol as an important modifier of disease risk. This has been exemplified by several studies showing the synergistic effect of alcohol consumption as a risk factor for the presence of significant fibrosis.[134], [135], [136] Most recently, data from the Liver Screen project showed that in approximately 30,000 individuals from the general population, 4.6% had elastographic signs of significant fibrosis.^137^ Elevated liver stiffness was strongly associated with obesity, type 2 diabetes and harmful drinking; moreover, in individuals with all three risk factors, the prevalence of significant fibrosis was 37.1%.^137^ Furthermore, studies have demonstrated the contribution of alcohol to liver disease progression in SLD.^10^^,^^11^^,^^25^^,^^138^^,^^139^ However, alcohol intake is notoriously difficult to quantify in a standardised and reliable manner, which has led to increasing interest in direct alcohol biomarkers, such as PEth. Although several studies have demonstrated that direct alcohol biomarkers can identify individuals with SLD who underreport their alcohol consumption,^140^^,^^141^ such markers, including PEth, provide only a limited, time-restricted (2-4 weeks) snapshot of drinking behaviour. This limitation is underscored by the substantial intra-individual variability in annual alcohol intake reported by Månsson *et al.*, as well as the observation by Israelsen *et al.* that more than one in four individuals with SLD transition between alcohol-use subcategories over time.^132^^,^^133^ Hence, as stated by the Basel consensus: “a single determination of PEth concentration may not give a full insight into drinking patterns”.^67^

Much of the existing literature on alcohol and SLD has focused on categorising alcohol consumption into abstinent/low, moderate, and excessive use. While such categorisation is methodologically convenient and often necessary in research settings, it may oversimplify a biologically continuous exposure. Alcohol consumption should instead be conceptualised as a continuous variable, analogous to BMI or HbA1c. A parallel can be drawn to the pre-elastography era, when histological cirrhosis stages encompassed heterogeneous patient populations with markedly different prognoses.

In a recent study by Torp *et al.*, individuals with low self-reported alcohol consumption and low AUDIT-C scores rarely exhibited PEth concentrations >80 ng/ml, whereas those reporting excessive alcohol intake frequently had high PEth levels, rendering PEth measurements redundant in approximately 40% of cases.^22^ Based on these findings, the authors proposed PEth thresholds of <80 ng/ml for MASLD, 80–200 ng/ml for MetALD, and >200 ng/ml for ALD. These thresholds are discordant with earlier proposed cut-offs of 20 or 35 ng/ml,^142^^,^^143^ values that may be exceeded even after moderate alcohol consumption.^103^ Rather than relying on dichotomous thresholds, future studies should focus on narrower PEth strata and evaluate their associations with clinical outcomes, including hepatic decompensation and liver-related mortality. Taken together, these findings have direct implications for how PEth should be integrated into the MASLD–MetALD–ALD framework, which was largely developed using self-reported alcohol intake. Self-reported consumption and biomarker-derived exposure should be viewed as complementary rather than interchangeable measures. A mechanical re-labelling of individuals with modestly elevated PEth values as MetALD would risk markedly inflating the prevalence of MetALD without clear evidence of improved risk stratification or clinical utility. Given that current alcohol cut-offs were derived from cohorts prone to underreporting, it is likely inappropriate to apply identical numerical thresholds to PEth-derived exposure. Instead, PEth should primarily serve to contextualise risk and inform longitudinal assessment, rather than act as a stand-alone determinant of disease category in routine clinical practice.

Furthermore, although accurate quantification of alcohol intake is important, the purpose of doing so warrants reflection. From a patient perspective, rigid categorisation may be of limited relevance, even though alcohol intake clearly carries prognostic implications for liver-related morbidity and mortality. Rather, the primary objective should be to identify and mitigate modifiable risk factors. In patients with SLD, alcohol is often only one of several contributors to disease progression. A holistic, nonjudgmental approach is therefore essential, with comparable efforts devoted to addressing alcohol consumption, cardiometabolic risk factors, and obesity in particular. Patients should have equitable access to pharmacological and lifestyle interventions targeting cardiometabolic dysfunction as well as those aimed at reducing harmful alcohol use.

Importantly, excessive alcohol consumption is frequently associated with stigma and shame. While recall bias is well recognised, intentional underreporting is likely equally prevalent. This became particularly evident in Sweden following the recommendation by the Swedish Transport Agency that physicians disclose PEth values exceeding 210 ng/ml. As a consequence, several patients lost their driving licenses without a clear understanding of the underlying rationale.^144^ In our clinical setting, PEth has long been included as a routine test for patients with chronic liver disease. During this period, however, we have observed increased patient hesitancy regarding blood sampling and a notable shift toward lower PEth values, with several individuals previously exhibiting moderate levels subsequently presenting values <35 ng/ml. This situation sparked a national debate, highlighting the need to distinguish between alcohol dependence and excessive alcohol consumption – two related but distinct clinical entities.

Nevertheless, in a research context, the impact of alcohol on chronic liver disease cannot be ignored. Studies in SLD, whether retrospective, cross-sectional, or prospective, should therefore routinely incorporate direct alcohol biomarkers or, at a minimum, validated instruments such as AUDIT or AUDIT-C. Importantly, it is increasingly difficult to justify evaluating novel therapeutic agents in clinical trials without systematic assessment of alcohol consumption, ideally using direct biomarkers both at baseline and during follow-up.

## Abbreviations

ADH, alcohol dehydrogenase; ALD, alcohol-related liver disease; AUDIT(-C), Alcohol Use Disorders Identification Test(-Consumption); BAC, blood alcohol concentration; CDT, carbohydrate-deficient transferrin; CYP2E1, cytochrome P450 2E1; EtG, ethyl glucuronide; EtS, ethyl sulfate; FAEEs, fatty acid ethyl esters; LC–MS/MS, liquid chromatography–tandem mass spectrometry; MASLD, metabolic dysfunction-associated steatotic liver disease; MetALD, metabolic dysfunction-associated alcohol-related liver disease; PEth, phosphatidylethanol; SLD, steatotic liver disease; TLFB, timeline followback.

## Authors’ contributions

Drafting of manuscript: all authors. Critical revision: all authors. All authors approved the final version of the article including the authorship list. Guarantor of the article: Patrik Nasr.

## Declaration of generative AI and AI-assisted technologies in the manuscript preparation process

During the preparation of this work the authors used Microsoft’s M365 Copilot in order to improve language and readability. After using this tool, the authors reviewed and edited the content as needed and takes full responsibility for the content of the published article.

## Financial support

PN was supported by ALF Grants (RÖ-1033510), Region Östergötland, the Wallenberg Center for Molecular Medicine, Linköping University, Mag-Tarmfonden, and the Lion's Research Grant, Linköping, Sweden, Bengt Ihre Foundation, Sweden (SLS-959713), 10.13039/501100007687Swedish Society of Medicine Project Grant (SLS-960925). ME was supported by ALF Grants, Region Östergötland, (RÖ-962520), Bengt Ihre Foundation, Sweden (SLS-960485), 10.13039/100010805Medical Research Council of Southeast Sweden (FORSS-995187), and Ruth and Richard Juhlin Foundation (2024-00262, 2025-00330).

## Conflicts of interest

PN has served as an advisory board member for Boehringer Ingelheim. ME has received lecture fees from Novo Nordisk, Mediplast, served as an advisory board member for Ipsen and received research grants from AstraZeneca and Takeda Pharmaceutical.

Please refer to the accompanying ICMJE disclosure forms for further details.
